# Association of Vaginal Microbiota With the Genitourinary Syndrome of Menopause Across Reproductive Stages

**DOI:** 10.1093/geroni/igaa057.554

**Published:** 2020-12-16

**Authors:** Michelle Shardell, Patti Gravitt, Jacques Ravel, Anne Burke, Rebecca Brotman

**Affiliations:** 1 University of Maryland School of Medicine, Baltimore, Maryland, United States; 2 Johns Hopkins Medical Institutions, Baltimore, Maryland, United States

## Abstract

The genitourinary syndrome of menopause (GSM) is a collection of signs and symptoms related to estrogen decline that involves physical changes to the vulva, vagina, and lower urinary tract. GSM signs and symptoms may occur during any reproductive stage but are most common during menopause. Vaginal microbiota, particularly Lactobacillus spp., protect the female genital tract from multiple conditions; however, Lactobacillus spp. abundance declines during menopause. We aimed to determine the longitudinal association of vaginal microbiota with GSM signs and symptoms across reproductive stages. In a two-year cohort study comprising 750 women aged 35-60 years who contributed 2111 semiannual person-visits, low-Lactobacillus spp. vaginal microbiota communities were observed at 21.2% (169/798), 22.9% (137/597), and 49.7% (356/716) of person-visits among pre-, peri-, and post-menopausal women, respectively (p<.001). After covariate adjustment, low-Lactobacillus spp. communities characterized by high Atopobium and Megasphaera relative abundance were associated with vulvovaginal atrophy relative to high-Lactobacillus spp. communities dominated by L. crispatus (OR[Odds Ratio]=3.04, 95% Confidence Interval[CI]=1.02-9.06) among post-menopausal, but not among peri- or pre-menopausal women. Also, post-menopausal women with low-Lactobacillus spp. communities reported decreased libido (OR=1.79, 95%CI=1.04-3.12) and vaginal dryness (OR=1.61, 95%CI=0.89-2.90) more frequently than their counterparts with high-Lactobacillus spp. communities, but not among peri- or pre-menopausal women (p for interaction<.05). Specifically, low-Lactobacillus spp. communities characterized by high Atopobium and Megasphaera relative abundance were related to both decreased libido (OR=2.82, 95%CI=1.11-7.14) and vaginal dryness (OR=3.50, 95%CI=1.18-10.44) compared with high-Lactobacillus spp. communities dominated by L. gasseri/L. jensenii. Vaginal microbiota, particularly Lactobacillus spp., and menopause may synergistically influence GSM.

